# Perspective: Defining Carbohydrate Quality for Human Health and Environmental Sustainability

**DOI:** 10.1093/advances/nmab050

**Published:** 2021-05-05

**Authors:** Rebekah Schulz, Joanne Slavin

**Affiliations:** Graduate student, University of Minnesota, St. Paul, MN, USA; Department of Food Science and Nutrition, University of Minnesota, St. Paul, MN, USA

**Keywords:** quality carbohydrates, added sugar, dietary patterns, nutrition, environmental sustainability

## Abstract

Plant foods are universally promoted for their links to improved human health, yet carbohydrate-containing foods are often maligned based on isolated, reductionist methods that fail to assess carbohydrate foods as a matrix of nutrients and food components. Currently accepted positive carbohydrate quality indices include plant food, whole-grain content, and dietary fiber, while negative health outcomes are linked to high intakes of added sugar and high glycemic index. More recently, negative health aspects have been linked to ultra-processed foods, which are often high in carbohydrates. Yet, carbohydrate staples such as grains and dairy products are both enriched and fortified, resulting in these carbohydrate foods containing important nutrients of concern such as dietary fiber, potassium, vitamin D, and calcium. This Perspective analyzes carbohydrate metrics used in dietary guidance and labeling and finds limitations in accepted indices included in standardized quality carbohydrate definitions and also proposes additional indices to benefit both human and environmental health. As nutrition recommendations shift away from a single-nutrient focus to a more holistic dietary pattern approach that is flexible and adaptable for each individual, it is necessary to determine the quality components that make up these patterns. This review concludes that current approaches that demonize staple carbohydrate foods do little to promote the recommended patterns of foods known to improve health status and reduce disease risk.

## Introduction

Throughout the past decades there has been an ongoing debate concerning what constitutes a healthy diet in order to inform healthy dietary patterns that positively impact both human health as well as the environment. With nutrition research evolving through the years and re-evaluating prior knowledge, while it can be difficult to definitively establish nutrition population guidelines it is important to continue to refine these guidelines because what we eat is integrally related to our short- and long-term health as well as noncommunicable disease morbidity and mortality (1). The recent marked impacts of the coronavirus disease 2019 (COVID-19) pandemic, which have highlighted the intrinsic link between what we eat and our immunity (2); the trend of overweight and obesity in the United States, often attributed to poor nutrition intake and knowledge and greatly impacting health care costs, quality of life, and health care outcomes (3); and thus following suit, the call to follow a more plant-based dietary pattern that will help mitigate both health issues related to food intake as well as food production's intensive negative impact on the environment, all highlight the need for well-defined nutrition quality indices.

**FIGURE 1:**
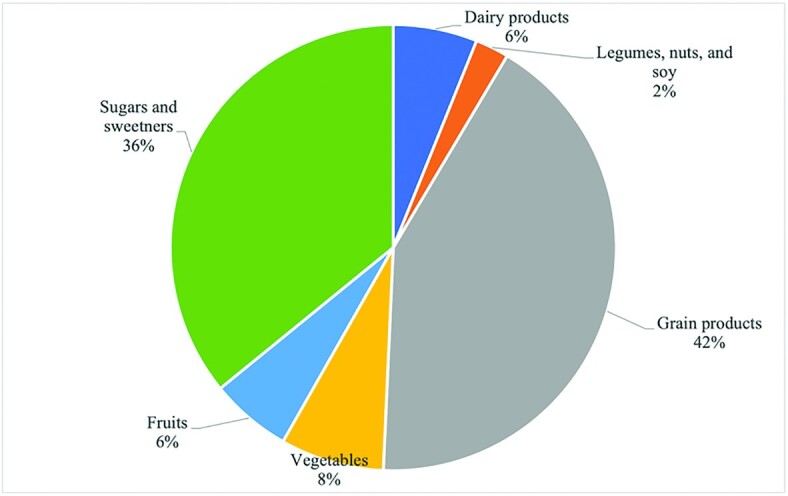
Carbohydrates contributed across food groups per capita per day (4).

Of all the macronutrients, carbohydrates have been stigmatized when it comes to health impacts; however, they make the highest percentage of intake worldwide, so defining quality indices is paramount for their inclusion in diet recommendations for human health and sustainability. However, for various reasons, including the imprecision of most human nutrition research trials, as well as the differential effects that diet can have on individuals based on genetics and lifestyle, identifying “healthy” dietary patterns is complex. A growing push to include additional metrics to create sustainable systems, such as the impact of food production and waste on the environment and food security, as well as economic and cultural factors, can further complicate the issue (5). Nevertheless, the effort to identify quality dietary patterns that can be produced and distributed in an equitable, sustainable manner is necessary for human and environmental health both for the short and long term.

This review briefly addresses the strengths and weaknesses of current methods used to assess carbohydrate quality, proposes additional indices to include in a standardized quality carbohydrate definition, and defines research questions for further exploration.

## The Goal: Defining Carbohydrate Quality

### The chemistry of carbohydrates

Foods that are high in carbohydrate are the basis of our diets, with usual intake of carbohydrates providing more than half of our calories. This is consistent with recommendations from the DRIs, which state that 45–65% of our calories come from carbohydrates (6). Carbohydrates include a diverse group of compounds, sugars, starches, and dietary fiber. Sugars are found naturally in fruits and dairy products, while starches are found in bread, cereals, and starchy vegetables (**Figure 1**) (4). Sugars can also be extracted from concentrated sources such as sugar beets or sugar cane or starches can be hydrolyzed to convert starch to sugars, such as production of sugars from corn. Digestible carbohydrates provide 4 kcal/g.

Nondigestible polysaccharides are deemed dietary fiber. Thus, starch that is resistant to digestion is considered resistant starch and is included in dietary fiber. As a result, dietary fiber is a diverse mixture of polysaccharides that share the physiological trait that they are not digested and absorbed in the upper digestive tract and can be fermented in the gut. The degree of fermentation of fiber varies, with some fibers being extensively fermented while other fibers, such as purified cellulose, are poorly fermented (7). The products of this fermentation, SCFAs and changes in the microbiota, have health benefits that are linked to fiber's role in the prevention of disease (8).

### Dietary patterns and carbohydrate adequacy

Dietary patterns are defined as the quantities, proportions, variety, or combinations of different foods and beverages in diets and the frequency with which they are habitually consumed (9). The shift to look at food through the lens of dietary patterns versus individual foods is a move to help assess day-to-day dietary habits of consumers, as well as make relevant recommendations that are holistic and not reductionist.

The Dietary Guidelines for Americans (DGA) have been published since 1980 and are released every 5 y jointly by the USDA and Department of Health and Human Services (HHS). The goal of combining the expertise of USDA and HHS was to ensure that the dietary recommendations could be more consistent and less confusing to practitioners and the public. As the DGA affect all nutrition policy in the United States, it is crucial that they are well supported by science.

**TABLE 1:** Current DRIs for carbohydrates for the RDA, AMDR, AI, and MyPlate^1^

	Carbohydrate recommendations
AMDR (10)	45–65% of calories in the diet should come from carbohydrates
DGA (11)	<10% of calories from added sugar
RDA for adults and children greaterthan= 1 y (10)	130 g/d
RDA for pregnant women (10)	175 g/d
RDA for lactating women (10)	210 g/d
AI, 0–6 mo (12)	60 g/d
AI, 6–12 mo (12)	95 g/d
AI for fiber (6)	14 g fiber/1000 calories
MyPlate (13)	Make half your plate fruits and vegetables Make half your grains whole grains
MyPlate (13)	Eat 3–8 1-ounce equivalents of whole grains/day
MyPlate (13)	Eat 1–2 cups of fruit/day
MyPlate (13)	Eat 1–3 cups of vegetables/day

^1^AI, Adequate Intake; AMDR, Acceptable Macronutrient Distribution Range; DGA, Dietary Guidelines for Americans.

The DRIs for carbohydrates, as determined by the DGA, include an RDA and an Acceptable Macronutrient Distribution Range (AMDR) (10). The RDA for carbohydrates is 130 g/d for adults and children aged ≥1 y and is based on how many sugars and starches the brain needs for an adequate supply of glucose. This amount increases to 175 g/d for pregnant women and 210 g/d for lactating women based on increased needs. The AMDR is 45–65% of total calories/d for adults and children ≥1 y and is not determined for infants <1 y. The AMDR minimum is greater than the RDA and was calculated based on epidemiological and not experimental evidence to avoid increased risk of obesity with low-carbohydrate, high-fat intakes and the upper limit to decrease the risk of chronic diseases and allow for the adequate intake of other nutrients. Adequate Intake (AI) levels have been set for infants aged 0–6 mo (60 g/d) and 6–12 mo (95 g/d) (12).

In addition, reference values have been set for specific categories of carbohydrates such as fiber and added sugar based on epidemiological studies examining the correlation between their consumption and the risk for development of a particular disease outcome (6). The AI for fiber was established at 14 g/1000 kcal due to prospective cohort studies that showed protection against cardiovascular disease with higher intakes of dietary fiber (6). Definitions and regulations continue to evolve for dietary fiber, but values for total dietary fiber are required on the Nutrition Facts panel in the United States with the current Daily Value (DV) for dietary fiber set at 28 g/d (7).

With regard to added sugar, the DGA suggest that <10% of calories should come from added sugars, a target based on food-pattern modeling and national data to keep nutrient needs within calorie limits as well as to prevent diseases correlated with excessive sugar intake (11). Labeling is required for total sugars, added sugars, and total carbohydrate on the Nutrition Facts panel. Total carbohydrate is generally measured “by difference,” while total sugars are measured by accepted chemical methods (8).

Further recommendations from the USDA MyPlate, which makes recommendations based on how a typical plate or meal should look, suggest that children and adults consume half of their plate as fruits and vegetables, half of their grains as whole grains, and then specific serving amounts of grains, fruits, and vegetables (**Table 1**) (13).

Overall carbohydrate intake is adequate in the United States. According to NHANES data from 1999 to 2016, the estimated percentage of energy intake from total carbohydrates decreased from 52.5% to 50%, with an increase both in the consumption of high-quality carbohydrates and plant proteins (14). However, a high intake of low-quality carbohydrates, often defined as those found in processed foods, snack products, and carbonated beverages contribute to 42% of energy intake. As a result, despite these references and recommendations, Americans are still consuming large amounts of low-quality carbohydrates, furthering the need for a better quality carbohydrate definition and indices.

### History of carbohydrate consumption

Carbohydrates have traditionally been the largest source of energy for much of the world's populations due to their agricultural abundance and economic affordability and, to this day, still account for more than three-quarters of global crop production (11, 15, 16). They have recently been lauded for the fact that they are one of the most sustainable food groups for climate health and a move to plant-based dietary patterns would mitigate climate change (17).

However, regarding human health, carbohydrates have served their time as a vilified food group, where, along with fat, they were labeled as a main contributor to the obesity epidemic and its links to cardiometabolic-related diseases such as type 2 diabetes and cardiovascular disease and mortality (18–23). As fat has moved from being vilified to gaining greater acceptance in healthy dietary patterns, carbohydrates seem to be the last remaining food group to be stigmatized as a negative contributor to health. This is partially due to the popularity of a proposed “carbohydrate-insulin model,” which suggests that an overabundance of carbohydrate intake leads to hyperinsulinemia, and therefore endocrine dysregulation, causing energy to be shunted away from metabolically active tissue such as muscle and into adipose tissue (24). The model proposes that this then leads to increased dietary intake and decreased physical activity to compensate for this “cellular internal starvation,” resulting in weight gain and increased risk downstream for cardiometabolic-related diseases. Based on this model, studies have examined whether low-carbohydrate diets will produce superior results in weight loss as compared with high-carbohydrate diets following the logic of decreased insulin concentrations in the body, but they have failed to realize these findings.

One such study, utilizing carefully controlled, randomized, inpatient feeding, did not show the expected results of decreased carbohydrate intake, such as increased physical activity and thus lower body weight as was supposed with this “carbohydrate-insulin model,” suggesting that something else is at play besides carbohydrate quantity (25, 26). However, many critics of this study suggest that the results are unfounded and more research is needed in this area before further conclusions can be made. While this may be true, a meta-analysis looking at low-carbohydrate diets for weight loss found that there was no significant difference in weight loss between low-carbohydrate and low-fat diet groups, rather it was the method of long-term adherence to their specific protocol by which participants were able to lose weight and keep it off, again suggesting that something more is involved than quantity of carbohydrates in the diet (27).

As a result, the focus on carbohydrate quantity is now being shifted instead to that of carbohydrate quality as an indicator associated with the development of noncommunicable diseases. Nutrient quality is often defined both in terms of its impact on a person's physical health, growth, development, and reproduction, as well as psychological or emotional well-being (28). Total nutritional quality, defined using algorithms like the Overall Nutritional Quality Index (ONQI), a nutritional rating system that ranks foods based on nutritional quality factors such as saturated fat, vitamins, minerals, and the quality of protein and fat, correlates with health outcomes, including total chronic disease burden and all-cause mortality (29, 30). Specifically with regard to carbohydrates, the Global Burden of Disease Project emphasizes that the low dietary intake of quality carbohydrates is 1 of the 14 significant dietary risk factors in the United States that increase premature morbidity and mortality, accounting for one-third of the cases of premature morbidity and mortality (11).

### Defining carbohydrate “quality” is not as easy as it sounds

For any one of the macronutrients, it can be difficult to assign a linear metric for quality, but carbohydrates seem to have received little attention when it comes to a scientific, evidence-backed, nonreductionistic methodology for defining their quality. Protein and fat have both been analyzed and a framework for quality has been established—for example, the Protein Digestibility Corrected Amino Acid Score (PDCAAS) and newly updated Digestible Indispensable Amino Acid Score (DIAAS) methods for protein quality assessment, methods that analyze amino acid content and assign a quality ranking as a result (31). However, unlike protein, carbohydrates are not essential for human survival, evidenced by populations surviving with no or limited intake, and therefore no straightforward formula exists for assessing their quality (32, 33). In addition, there are many types and categories of carbohydrates, with little homogeneity in structure, composition, or their impacts on various health and disease matrices, further adding to the complexity.

Some carbohydrate sources have been given a “healthy halo,” such as whole grains, nonstarchy vegetables, and legumes, and indeed are sensible foods to consume due to their favorable impact on blood sugar response, satiety, the gut microbiome, and overall human health (34, 35). However, in some instances, based on method of preparation and other factors, even data regarding these sources are not as straightforward to evaluate as one might think, leading to some foods receiving a misplaced halo. Other sources, primarily refined grains and foods with added sugars, are generally considered lower-quality carbohydrates due to their health impacts. Based on available evidence it is not difficult to agree that these foods should not be major dietary staples. As a result, carbohydrate quality has only thus far been based on simplistic and reductionistic tools like glycemic index (GI) or fiber content, and a well-defined, holistic framework that takes into account all of the indices of carbohydrate quality has not yet been formulated (11, 36).

### What metrics have been used to determine carbohydrate quality?

Historically, researchers and health professionals have equated carbohydrate quality with a single metric like glycemic index (GI), whole-grain ratio, fiber content, or percentage of added sugar, single markers that have often been used in isolation rather than in a combined method to assess quality, with no standardized algorithm to establish a universal method (34).

**FIGURE 2:**
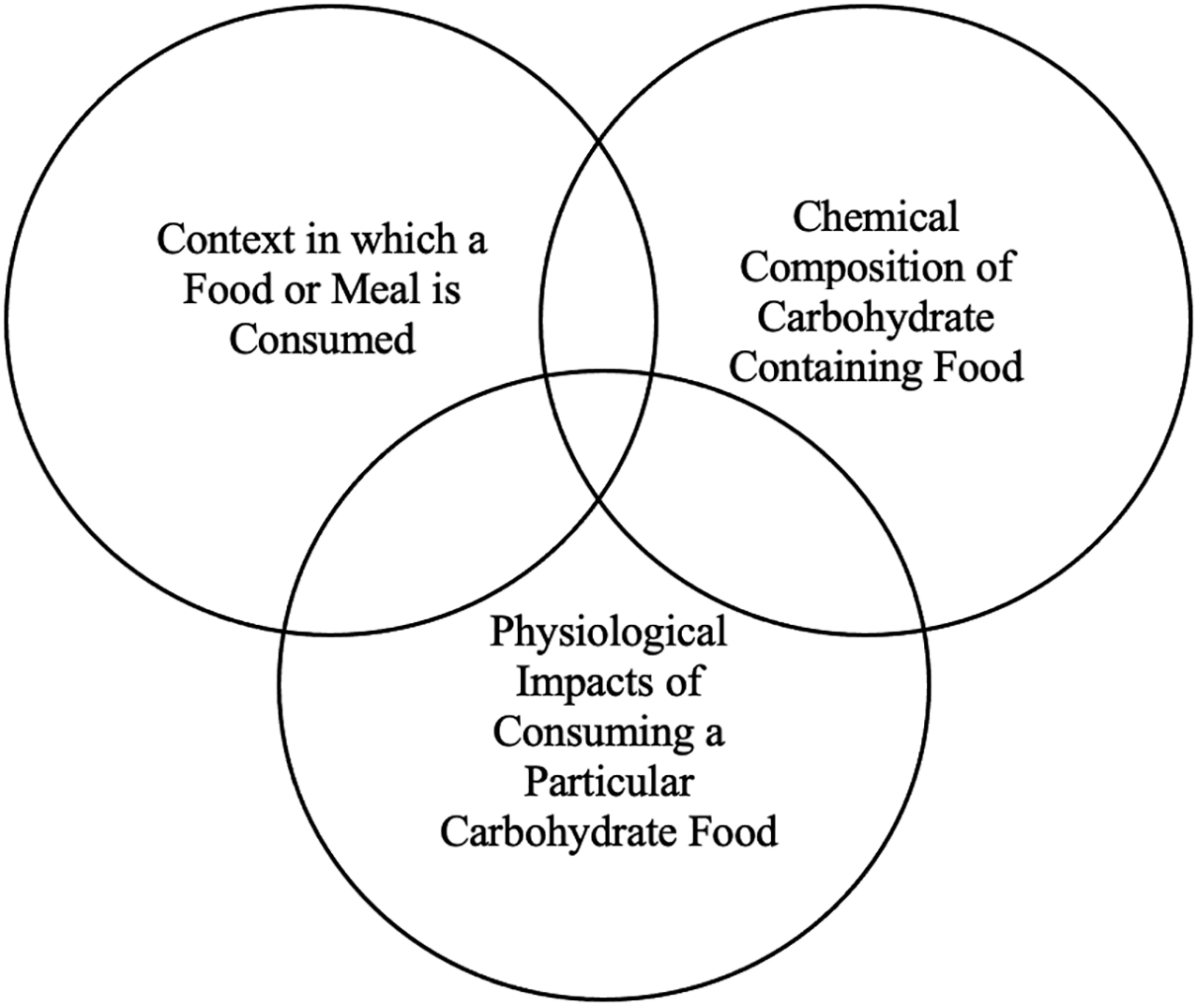
Grouping of various criteria on what designates a quality carbohydrate (35) including context in which a food or meal is consumed, chemical composition of carbohydrate-containing food, and physiological impacts of consuming a particular carbohydrate food.

A recent paper based on inputs from various carbohydrate nutrition and food science experts identified upwards of 20 factors that could be applied in determining carbohydrate quality (37). The paper suggested that these quality criteria could fall into 3 major physiological/sociological categories: context in which a food or meal is eaten, chemical composition of the carbohydrate-containing food, and physiological impacts of consuming a particular carbohydrate food (**Figure 2**). If a food fell at the center, meeting all the various criteria expressed by the grouping, it would be a well-rounded, quality carbohydrate.

**FIGURE 3:**
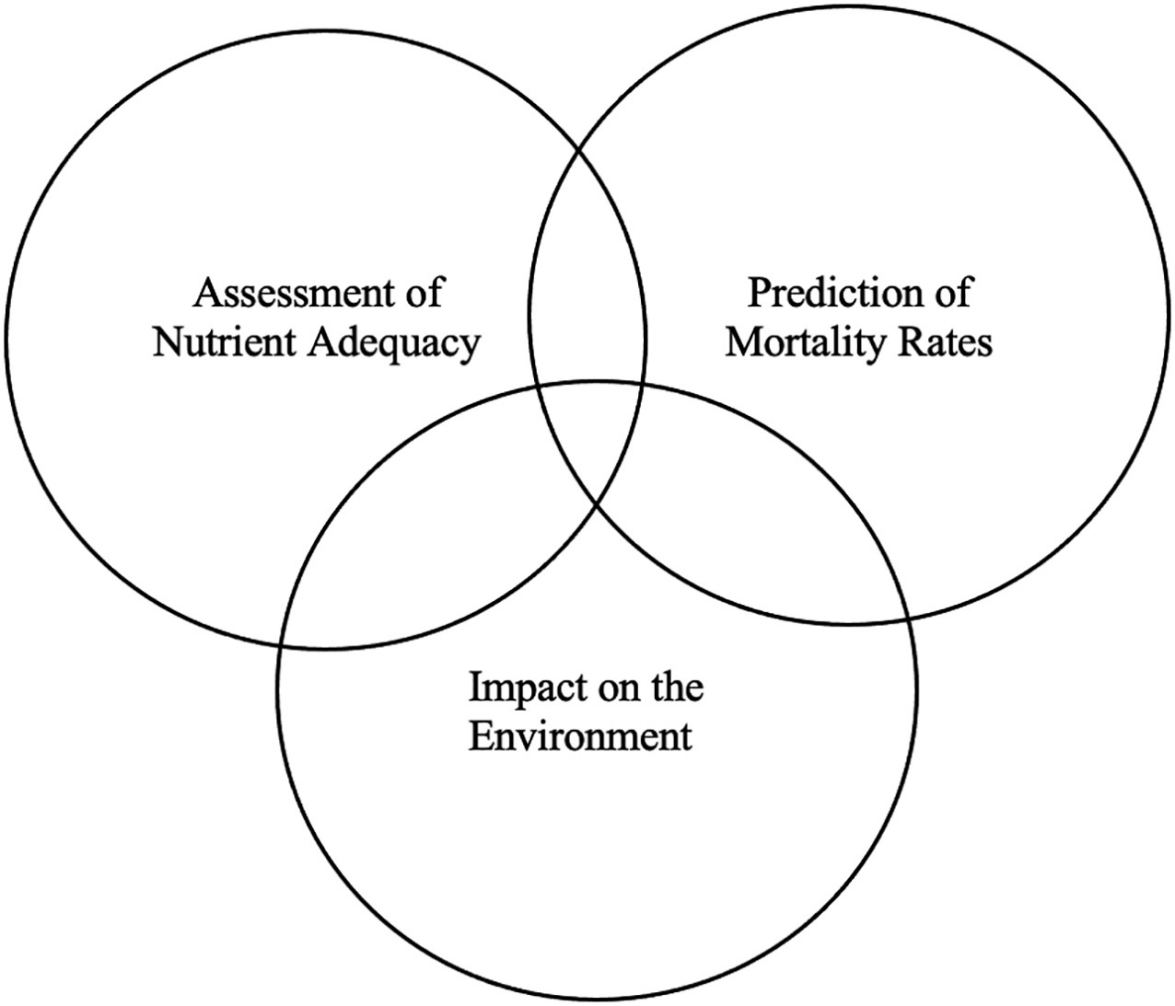
EAT-Lancet Commission on a system to determine quality carbohydrates including the spheres of assessment of nutrient adequacy, prediction of mortality rates, and impact on the environment (17).

In addition, the EAT-Lancet commission, which defines parameters for healthy diets and sustainable food production, suggested environmental impact as a key component of carbohydrate quality, establishing a different model that takes more of an environmental and physiological approach (**Figure 3**) (17).

As can be seen in Figures 2 and 3there is no straightforward or agreed-upon method or algorithm to determine quality carbohydrates. Furthermore, within each of these groupings, there is a broad list of factors that could be indices for quality, such as the context in which a food or meal is habitually consumed, overall lifestyle of the consumer, food form, food-preparation method, fiber/sugar/protein content of the food in question, at what time of the day the food is consumed, impact of the food on various biomarkers, as well as cost and availability issues. **Table 2** lays out the indices that could be used as quality indicators, currently accepted carbohydrate quality indices in the United States, and which carbohydrate-containing foods these indices have informed as a quality carbohydrate as a result.

**TABLE 2:** Comparison of debated quality carbohydrate indices with currently accepted quality carbohydrate indices that then inform foods that are accepted as quality carbohydrate-containing food sources

Defined carbohydrate quality indices	Currently accepted and utilized indices	“Quality” carbohydrate-containing foods based on currently accepted indices
• Percentage/ratio of fiber• Fiber type• Starch type and properties• Resistant-starch content• Rate of starch digestion• Sugar content• Carbohydrate digestibility fractions• Nutrient density• Micronutrients/phytonutrient content• Other factors that affect rate of absorption• Protein content• Protein quality• Whole-grain composition• Environmental sustainability• Prebiotic composition• Glycemic index and load	• Whole-grain content• Fiber content• Percentage of added sugar• Glycemic index	• Whole grains (i.e., rice, oats, wheat, barley, corn, rye)• Nonstarchy vegetables• Nuts and legumes• Pulses

Based on all of this information, for a consumer to make a quick choice about a carbohydrate without a formula or algorithm would be impossible, again suggesting the need for a more holistic and streamlined way to determine carbohydrate quality.

The next section will look more in depth at the indices that have historically been used to indicate carbohydrate quality, their development, and whether they still stand as a viable and relevant option to be included in a potential quality carbohydrate formula.

### Currently accepted carbohydrate quality indices

GI, glycemic load (GL), whole-grain content, fiber, and added sugar have long been accepted and utilized as indicators of carbohydrate quality. **Table 3** compiles the definitions, pros and cons, discussion, and conclusion on their use in a future algorithm for each of these indices.

**TABLE 3:** Definitions of currently accepted indices for determination of quality carbohydrate-containing foods along with the pros and cons, discussion, and conclusion on including them in a future carbohydrate quality algorithm^1^

Index	Definition	Pros	Cons	Discussion	Conclusion
Glycemic index (GI) and glycemic load (GL)	GI indicates the blood glucose response from consuming a particular carbohydrate-containing food as compared with a carbohydrate-containing reference food, typically glucose or white bread (38)	Beneficial for diabetes patients as a way to effectively control blood sugar (38).Potentially translatable for prevention and treatment of other diseases such as obesity, cancer, and heart disease (39).Potentially beneficial when used in combination with other nutritional quality indices to determine nutrient quality (29)	Labile marker that is alterable based on the effect of foods eaten together, preparation of that food, and time of day that the food is eaten (37, 40, 41).GI and GL are expressed in isolation while real-life dietary intake is not that of single nutrients, but food consumed within a matrix that involves interactions between different nutrients, the nutrients and the food form, and the foods and dietary patterns that contain those foods (42)	First publication on GI was introduced in 1981 as a ranking system of various carbohydrates based on their impact on postprandial glycemia and was originally intended for diabetes patients (41, 43).GL was developed shortly thereafter to describe the amount of carbohydrates in a portion of food and its corresponding increase in blood glucose concentrations. Although originally indicated for diabetes patients, GI and GL have been recommended for translation to other disease populations such as obesity, cancer, and heart disease for treatment and prevention of these diseases, with the need for impact assessment (39).The ICQC, an international summit held by experts in carbohydrate research, identified the important role that postprandial glycemia plays in health outcomes and therefore indicated GI as a valid index to measure carbohydrate quality (44).A review on a series of meta-analyses on GI and GL agrees with the ICQC, but with the caveat that many low-GI and -GL foods tend to be more energy dense and higher in undesirable fats, unfavorable nutritional characteristics for population health and therefore not indices to use in isolation of other factors (38). However, as exemplified by minimally processed and naturally occurring carbohydrates in grains, fruits, and vegetables, which are naturally low in GI and GL, the basis for their inclusion in reference diets is based on their additional qualities	A variety of systematic reviews and meta-analyses conclude that dietary fiber and whole-grain content would be more beneficial in determining carbohydrate quality over using GI or GL due to the mass quantity of studies correlating fiber and whole-grain content with improved health outcomes in comparison to GI and GL (35). It is noted that GI and GL can be useful for specific diseases such as T2D or as a complement to other carbohydrate quality markers but may not be as translatable to carbohydrate quality in isolation due to its reductionist approach (43, 45)
Whole grain (WG) and WG food	WGs are grain with specific components and proportions of endosperm, germ, and bran (46).WG food refers to the quantity of grain in a certain food product	The mechanisms of action by which WGs benefit human health is still debated, but the accolades of their quality are due to their provision of fiber, phytonutrients, vitamins, and minerals (47).Systematic reviews analyzing WGs effect on markers like weight loss/maintenance, improved glycemic control, blood lipids, and blood pressure show improvements in all of these markers but only when restricted to specifically oats and barley (48).Additional reviews have shown a positive effect of WGs on decreased cardiovascular disease incidence, diabetes incidence, and cardiovascular mortality, as well as all-cause mortality (34, 37)	Due to the variety in types of WGs as well as definitions of WG foods, it can be difficult to construct a consistent way to measure health outcomes from consumption and therefore elucidate correlations between health and WG consumption (49)	Since the rise of agriculture, WGs have been a key component of the human diet, with the most commonly consumed grains in the United States including wheat, oats, rice, maize, and rye, with wheat contributing the most to total intake (66–75%) (7).Recommendations around WGs were first implemented in the Dietary Guidelines in 2000 and have evolved over the years from “choose a variety of grains, especially whole grains” to “eat at least 3-ounce equivalents (oz-eq) of whole grain daily, and at least half of all grains consumed should be whole grains” in 2005–2010, with this messaging continuing in the 2020–2025 DGA guidelines (11, 50, 51).This messaging has seen a subsequent rise in WG consumption (52).There is debate about prioritization of fiber content over WG content as an indicator of quality and therefore health benefit, with research underway to determine the relation (46)	Despite the discrepancies in definitions of WGs, WG foods, and their corresponding health benefits, the knowledge of WG consumption as a benefit to human health is well established and therefore should be included as a carbohydrate quality index
Fiber	Total fiber consisting of dietary fiber and functional fiber, with dietary fiber composed of nondigestible carbohydrates and lignin that are intrinsic and intact in plants, and functional fiber as isolated, nondigestible carbohydrates that have beneficial physiological effects in humans (9, 53).Dietary fiber can be further broken down into soluble and insoluble fiber with different physiological benefits from each group	A series of systematic reviews have shown that high viscous soluble fiber from oats and barley has resulted in improved blood lipids, decreased systolic and diastolic blood pressure, and improved glycemic control, thereby decreasing the risk of various cardiometabolic diseases (54–56).Insoluble fiber consumption does not similarly result in lowered blood lipids, specifically from wheat bran, but diets rich in these insoluble fibers do benefit laxation (7, 39).An additional set of systematic reviews and meta-analyses indicated that fiber, irrespective of its food source, resulted in decreased incidence of diabetes and cardiovascular disease (34, 57, 58)	Due to debates about the labeling and measurement of fiber, as well as added fiber versus intact fiber, it can be difficult to consistently assess fiber content and type as related to health outcomes (7)	A recent paper examined a variety of carbohydrate ratios within carbohydrate-containing foods, with fiber as the consistent component to determine healthier foods and health outcome (37).While there is debate surrounding the specifics of fiber identities and therefore measurement and correlative abilities, fiber consistently appears to present itself as a valuable marker of quality	As a result, similar to WGs, fiber content seems to be an indicated and necessary metric to include in a quality carbohydrate formula
Added sugar	Added sugars are defined as those that are added to foods during processing, manufacturing, or preparation and therefore not naturally present in that food. This is in contrast to natural sugars, such as those found in whole fruit or dairy milk products	Epidemiologic and experimental evidence highlights added sugar and specifically sugar-sweetened beverages as a major public health concern when it comes to obesity (59, 60).The DGAC (61), a committee of nutrition science experts who analyze current data to make nutrition recommendations to the USDA and the HHS in their development of the DGAs, and additional studies further note that intake of added sugars, specifically sugar-sweetened beverages, also increases the risk of T2D, coronary heart disease, hypertension, elevated blood pressure, and stroke, and higher intakes of free sugars are linked to the development of dental caries in children and adults (59–63).An established added-sugar guideline allows for enforcement and regulation	Including added sugar as a metric is difficult for policy reasons due to the inability to analytically measure added sugar, as well as the differing standards between countries, and as a result, to attribute specific amounts to health outcomes (64)	Some studies suggest that, while sugar consumption is often correlated with these chronic diseases, this is not necessarily due to the sugar itself—for example, fructose in sugar-sweetened beverages—but rather the excessive consumption of sugar and other added-sugar–containing foods when compared with the same diets without excessive energy consumption from sugars (63). Therefore, studies suggest that it is not the presence of fructose or other added sugars themselves but rather the amount of added sugar consumed and the food matrix they are consumed within, highlighting this with the fact that whole fruit containing fructose often benefits health (65, 66).The DGA has taken different approaches over time to deal with empty calories or discretionary calories. Added sugars fit in this category as they add calories to food products but are not “intrinsic” in recommended carbohydrate foods. Thus, the sugars in fruits and milk are considered part of recommended dietary patterns, while added sugars are not. This has been a struggle over time to determine best practices to regulate and label added sugars as added sugars are always captured in total sugar chemical methods. So, if added sugars are required on a label, then those values must be calculated by established rules (64, 67, 68).The 2015 DGAC set a recommendation to limit intake of added sugars. The scientific basis of guideline recommendations of sugar intake has been extensively debated (67), with concerns about our inability to measure total sugar and added-sugar intakes in populations and in foods. But the 2015 DGA set a goal of 10% of calories coming from added sugars and the FDA included a DV for added sugars and a requirement for added-sugars labeling as part of the Nutrition Facts panel (67). The 2020 DGA are consistent with the 10% of calories as added sugars on its recommendation	Due to the agreed-upon stance regarding excessive consumption of added sugar and its impact on human health, percent added sugar seems a necessary indicator to include when developing a metric for quality carbohydrate designation

1 Data from references 7, 9, 11, 29, 34, 38–68. DGA, Dietary Guidelines for Americans; DGAC, Dietary Guidelines Advisory Committee; DV, Daily Value; HHS, US Department of Health and Human Services; ICQC, International Carbohydrate Quality Consortium; T2D, type 2 diabetes.

To further emphasize the need for a standardized metric for carbohydrate quality, the next section will look at real-food examples of how these 4 quality carbohydrate indicators have been used, highlighting how their use as isolated markers does not yield consistent findings for what designates a quality carbohydrate.

### Case studies: how current carbohydrate quality indices’ reductionistic approach leads to mistrust of nutritional recommendations for specific foods

#### Potatoes: the misunderstood carbohydrate

As mentioned, there are numerous examples of foods that are designated high or low quality based on limited, incomplete, or seemingly biased information. For example, potatoes have been consumed healthfully for centuries, serving as a main staple in various cultures, and yet in several recent studies have been paired with low-nutrient-dense products like sugar-sweetened beverages and sweets in a “low quality carb” bucket of foods to be avoided (17, 63). The recent EAT-Lancet report, which purports to be a global blueprint for how people should live and eat in the future to sustain both human and environmental health, strongly stresses the intake of more plant-based foods, but excluding potatoes and limiting their intake to only 39 kcal/d in their listed reference diet (66). This conclusion of potatoes as a low-quality carbohydrate appears to be based on the GI of some potato preparations and a handful of epidemiological studies that have identified potatoes as part of a dietary pattern associated with elevated disease risk (69–71). However, the claim that potatoes are low quality is solely based on the GI metric, which does not take into context the overall contribution that potatoes make to the carbohydrate and nutrient composition of the diet (72). Expanding on this, as potatoes contain 2 nutrients of concern, fiber and potassium, as well as vitamin C and resistant starch, and have been shown to result in greater subjective satiety when compared with equivalent portions of rice and pasta, it seems that more indices should be taken into consideration than simply the GI when determining potatoes’ level of quality as a source of carbohydrates (73, 74). In addition, potatoes are a cultural food with weighty implications both for cultural significance as well as food security in developing countries, something to also consider when analyzing this food source for its quality.

#### Nutritional “halo” foods: rice, pasta, and pulses may not be as nutritionally elite as touted

Conversely, carbohydrate-containing foods that have previously received a nutrition “halo” may not be as beneficial as suggested. There are data suggesting that rice and pasta produce a more adverse metabolic response than potatoes, whereas rice and pasta are often associated with higher quality (72). In addition, potatoes performed better with respect to the Healthy Eating Index score (75). While pulses do contribute many nutritional benefits, a study analyzed how the presence of bioactive compounds such as phytates, tannins, and polyphenols negated these benefits in the diet, indicating that processing is often key to achieving the most nutrition (76, 77). As a result, most pulses wear a nutritional “halo” but, in many instances, are not that nutritionally different than potatoes or some grain-based products.

Based on both of these real-world carbohydrate-containing food examples, it is evident that a holistic, quantitative approach is in order for more consistent and accurate determination of quality carbohydrates. This next section will examine further indices to include in a quantitative formula for carbohydrate quality.

## Proposed Carbohydrate Quality Indices

### Sustainability

A recent commission by EAT-Lancet brought together commissioners from 16 countries in various fields of human health, agriculture, political sciences, and environmental sustainability to determine scientific targets to reverse climate change via an altered global diet and environmental sustainability (17). The commission states that food production is among the largest drivers of global environmental change by contributing to climate change through greenhouse gas production such as carbon dioxide, nitrous oxide, and methane, as well as impacting biodiversity, animal welfare, nutrient leaching, and the use of chemicals. As a result, they state that “human diets inextricably link human health and environmental sustainability.” The Dietary Guidelines Advisory Committee (DGAC) also highlighted the link between food production, health, and sustainability, recommending dietary patterns that are able to satisfy all 3 parameters (78). Therefore, when designing quality indices for any food group, including carbohydrates, it is paramount to factor in the markers that would ensure that the diet would not only lead to better human health and the reversal and prevention of noncommunicable diseases but also meet the scientific targets to reduce climate change.

Based on these criteria, the EAT-Lancet commission proposed a reference dietary pattern that they suggest is adaptable and relevant for all global populations, both in terms of nutrient and energy adequacy as well as sustainability (17). This diet is plant based, enrolling carbohydrates as the greatest caloric contributor to the overall diet (≤60% of total kcal/d intake), specifically whole grains which included rice, wheat, corn, and other; protein sources primarily from plants, including soy foods; and legumes and nuts with only some fish and poultry additions in small quantities. This reference dietary pattern is inspired and rooted in traditional diets that are primarily plant-based, regional dietary patterns including grains, nuts, lean meats, and berries, and as a result, boast nutrient-rich and calorie-limited dietary profiles and result in lower BMI and rates of noncommunicable diseases (79).

In addition to the DGACs designation of the importance of carbohydrates as the basis for healthy dietary patterns, multiple meta-analyses looking at life-cycle assessments of various food products’ environmental impacts as well as current animal-based reference diets present grains, fruits, and vegetables as having the lowest environmental impact per serving as compared with meat from ruminants, which have the highest impact (78–80).

The 2015 DGAC also noted the importance of sustainable diets and listed the new US pattern, a Mediterranean-style pattern, and a vegetarian-style pattern, all heavily reliant on carbohydrates, specifically whole grains, vegetables, fruits, and nuts and legumes, as meeting criteria for environmental sustainability due to the focus on plant consumption (61).

However, the WHO's removal of support from the EAT-Lancet initiative draws question to the translatability of plant-based dietary patterns to all populations, specifically those in developing countries (81). The WHO was concerned that shifting dietary guidelines towards plant-based patterns would result in economic depression and the loss of millions of jobs connected to animal husbandry and processing, a loss of traditional diets that define and legitimize cultures, a loss of consumers’ freedom of choice, and nutritional deficiencies that would be dangerous to human health, all factors which are heightened and more detrimental for developing countries.

As a result, while it can be concluded that including sustainability as a metric for determining carbohydrate quality is essential due to the current state of the planet and the interdependent relations of food production, climate health, and human health, how we go about making recommendations for reference diets is equally important so as to ensure that all populations are treated with equity, sensitivity, and scientific backing when it comes to specific needs.

### Protein source

In addition, many carbohydrate-containing foods can also act as quality protein sources, allowing populations to shift towards plant-based dietary patterns while still meeting protein requirements, posing protein quality as a potential additional metric for determining the quality of carbohydrate-containing foods (82, 83). The United States currently uses the PDCAAS under the US FDA to rank the quality of protein sources, often resulting in animal-based protein ranking higher than plant sources due to digestibility and an amino acid profile that is considered aligned with human needs (84). However, the implication that animal protein is the best and only source of quality protein is misguided and not in alignment with the DGA statement on protein both for human and environmental health. In addition, contrary to the long-held belief that in order for plant protein to meet essential amino acid needs the consumer would need to combine complementary plant proteins in a meal, research now shows that simply eating a varied plant-based diet throughout the day will meet protein needs without the need to ensure that protein sources complement one another (85).

As a result, due to carbohydrates’ ability to provide protein and the need to shift dietary patterns towards plant-based and therefore carbohydrate-focused dietary patterns, the protein quality of these carbohydrate-based diets is paramount, indicating another metric to include in a standardized method for determining carbohydrate quality.

### Processed vs. ultra-processed

The extent of processing is another potential metric to consider for determining carbohydrate quality. Messages to avoid foods with >5 ingredients and avoid ultra-processed foods have been adopted in other countries (86, 87). While some studies have indicated a relation between increased consumption of ultra-processed foods and noncommunicable disease risk (88), and while the defined metrics appear to support whole foods and better food choices, they have no appreciation of the complexities of foods and the needs for additives for enhanced nutrition and food safety. Some of the benefits of the modern food system include lower postharvest food losses, safety, availability, convenience, choice, quality, and nutrient density (87). Scientific advisory committees for dietary guidance need experts beyond nutrition, including food science, food economics, agriculture, and food access, to advise on this topic (89).

## Bringing It All Together: Is an Algorithm the Solution to a Standardized Carbohydrate Quality Metric?

Now that a variety of indices of carbohydrate quality have been mentioned, both those that have already been accepted and others that could further benefit health and sustainability outcomes, it is important to mention how these indices might work together in a matrix, establishing a standardized way to define carbohydrate quality. More recently, attempts have been made to create models or equations that take into account multiple nutritional factors. For example, for overall diet quality, point systems like those used to decipher protein quality (90), the ONQI, and the graded coding method to analyze food-based dietary guidelines give or take away points for different components in a food and then indicate the quality of the food based on the sum of those components (27, 91). While not perfect, these models seem to be a step in the right direction toward identifying nutrient quality in a more holistic fashion.

**FIGURE 4:**
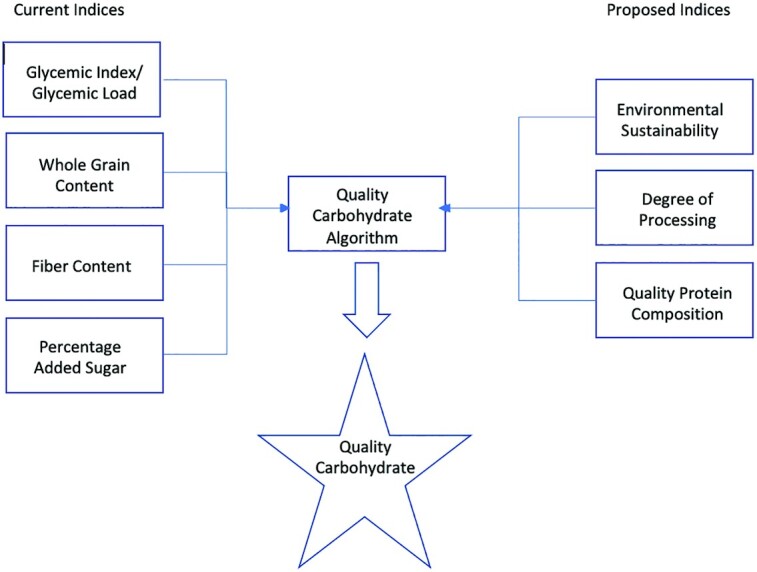
Current and proposed indices layered into an algorithm to produce a holistic, standardized quality carbohydrate metric.

We propose that a system similar to the aforementioned could work to decipher high- or low-quality carbohydrate sources—for example, an algorithm that takes into account the whole-grain, fiber, and added-sugar content, protein quality, processing, and environmental impact of a food, as well as ratios such as total carbohydrate to fiber and added sugar to fiber in a food, thereby using a holistic approach to assess quality. The result would positively impact both health and environmental outcomes and create consistent ways to measure intake across populations (**Figure 4**).

## Conclusions

As reference diets move from isolated nutrients to flexible dietary patterns, a greater need arises for the definition of the quality components that make up these patterns. This review addresses the need for a more linear, quantitative carbohydrate quality metric that takes into account already accepted indices of carbohydrate quality, including fiber, whole-grain content, and added sugar, as well as proposed indices of protein quality and environmental sustainability. Further research is needed on any additional metrics that could add to the carbohydrate quality definition as well as the best format and system for an algorithm to yield optimal results for environmental and human health. Overall, a holistic, nonreductionist approach to nutrition is key in addressing the complex needs of the population and planet, and defining carbohydrate quality using a standardized, holistic method is one more step in that direction for the health of the planet and its inhabitants.

## Notes

The authors reported no funding received for this work.

Author disclosures: JS has current grants from Taiyo and Barilla in the area of dietary fiber; she serves on the Scientific Advisory Boards for Tate and Lyle, Atkins Nutritionals, and the Quality Carbohydrate Council. RS reports no conflicts of interest.

Perspective articles allow authors to take a position on a topic of current major importance or controversy in the field of nutrition. As such, these articles could include statements based on author opinions or point of view. Opinions expressed in Perspective articles are those of the author and are not attributable to the funder(s) or the sponsor(s) or the publisher, Editor, or Editorial Board of *Advances in Nutrition*. Individuals with different positions on the topic of a Perspective are invited to submit their comments in the form of a Perspectives article or in a Letter to the Editor.

Abbreviations used: AI, Adequate Intake; AMDR, Acceptable Macronutrient Distribution Range; DGA, Dietary Guidelines for Americans; DGAC, Dietary Guidelines Advisory Committee; GI, glycemic index; GL, glycemic load; HHS, US Department of Health and Human Services; ONQI, Overall Nutrition Quality Index; PDCAAS, Protein Digestibly Corrected Amino Acid Score.
